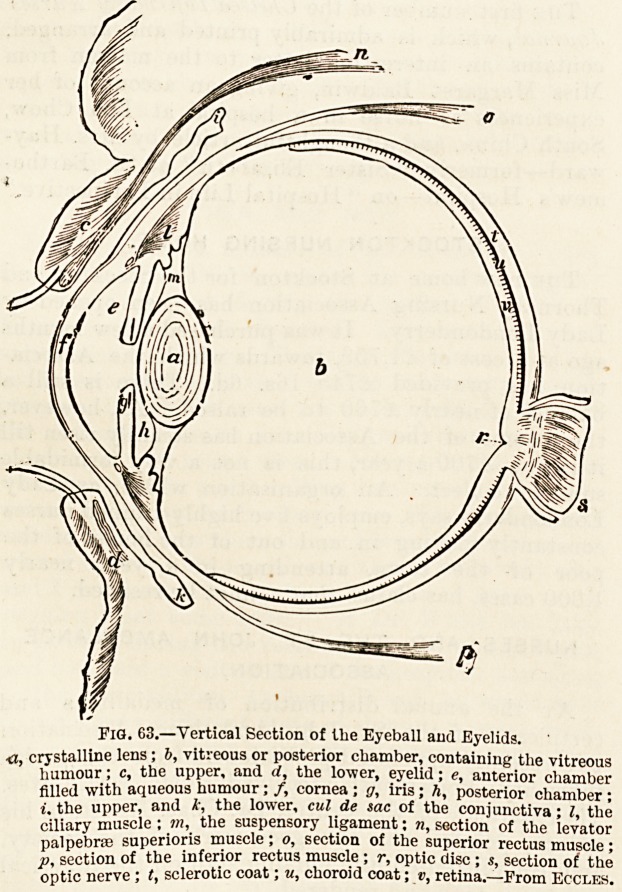# The Hospital. Nursing Section

**Published:** 1902-11-01

**Authors:** 


### The Hospital.

### Nursing Section.

Contributions for this Section of "The Hospital" should be addressed to the Editor, "The Hospital" Nursing Section, 28 & 29 Southampton Street, Strand, London, W.C.

No. 840.—Vol. XXXIII. *SATURDAY, NOVEMBER* 1, 1902.

### Notes on News from the Nursing World.

### THE ROYAL RED CROSS.

At the Investiture held by the King last Friday, several eminent members of the nursing profession were introduced to His Majesty, and received at his hands the decoration of the Royal Red Cross. The coveted honour was bestowed upon Miss Sidney Browne, matron-in-chief of Queen Alexandra's Imperial Military Nursing Service; Miss H. Wedgewood, matron of the Royal Free Hospital, Gray's Inn Road; Miss Ethel McCaul, Sisters E. C. Shannon, M. C. S. Knox, L. M. Stewart, of the Army Nursing Service, and E. C. Laurence, of the Army Nursing Service Reserve. The King also conferred the decoration upon Georgina Countess of Dudley, Mrs. Percy H. Johnston, Miss Daisy Brazier, Mrs. Emma Kate Francis, Miss H. Hogarth, Mrs. Sclater, and Mrs. Theodosia Bagot.

### THE PRINCESS LOUISE AT NOTTING HILL.

On Monday the Princess Louise, Duchess of Argyll, formally opened a nurses' home in Kenley Street, Notting Hill, which is intended as a memorial of the Diamond Jubilee of Queen Victoria, and has been erected under the auspices of the Kensington District Nursing Association. Her Royal Highness is President of the Association, and in that capacity she received an address from the chairman of the Jubilee Commemoration Committee asking her to accept the home and giving details of the history of the movement from its creation. It was stated that the home had only been occupied for three weeks, and that in the first 24 hours 220 persons applied for assistance. The Princess in reply said that it gave her great pleasure to be able to take over the building. She was sure that the nurses would help the district which needed their kind and elevating influence.

### LORD ROBERTS AND THE NURSES.

At the termination of the exhibition of drill by the children of the public elementary schools on Thursday last week, Lord Roberts pinned the South African medal on the breast of Private Fouch, of the Portsmouth Ambulance Corps, who had served in the late campaign under his command in the No. 6 Field Hospital. Lord Roberts then permitted Dr. C. Knott to introduce Miss Hall, the matron of the Portsmouth Union Infirmary, and five of the junior probationers who were present.

### ROYAL BRITISH NURSES' ASSOCIATION.

At the council meeting of the Royal British Nurses' Association on Friday last, a communication was read from Princess Christian consenting to open the sale of work on Tuesday, Dec. 2. It was also intimated that the Lord President of the Council had requested the Association to take steps to appoint a member of the Central Midwives' Board in accordance with the Act, and that in compliance with this request the committee had nominated Miss Dorothea Oldham to serve as its representative on the board. Miss Oldham was trained at Addenbrooke's Hospital, Cambridge. She has been matron of the Incurables' Home, Leamington, the General Hospital, Cheltenham, and the Shrewsbury Infirmary. For five years she was superintendent of the rural district branch of the Queen's Institute. During that time she had the superintendence of nearly 100 district nurses and midwives, whom she inspected, and from whom she received monthly reports, her duties as inspector extending for a long period, not merely to the rural districts of England, but also to Wales. In July, 1894, the council of the Queen Victoria Institute conferred on her, as superintendent of their rural district branch, the silver badge in recognition of her valuable services rendered to the institute in that capacity. Miss Oldham is at present matron of the Hospital for Epilepsy, Regent's Park, a post which she has held for the past five years. The nomination of Dr. Comyns Berkeley as medical honorary secretary was confirmed at the meeting, and it was signified that the name of Miss C. Leigh, matron of the Central London Sick Asylum, Cleveland Street, had been added to the list of ex-officio matrons on the General Council.

### TRAINED AND HALF-TRAINED NURSES IN RURAL DISTRICTS.

The correspondence which our statement that the towns, villages, and scattered districts of Cumberland in which the village nurses are engaged must remain at a disadvantage compared with other counties where fully-trained district nurses are employed has provoked, shows that the interest taken in the question is very great. Following a vindication of the village nurse by the Countess of Lonsdale, a not fully-trained nurse, writing from her own experience, insisted that a partially-trained nurse can never be as efficient as her fully-trained sister. These letters have been supplemented by communications from Lady Knightley, a District Nurse who accuses the Cumberland Nursing Association of trying to drive her away from the district, and, to-day, from the correspondent who originally made a similar charge, and who, in spite of Lady Lonsdale's denial, adheres to the assertion. We purposely leave the personal issue alone, and on the general question we desire merely to intimate our conviction that Lady Knightley has put the case in a nutshell. She shows most conclusively that it is well within the bounds of possibility to maintain a fully-trained nurse in any rural district, and, this being so, there cannot be the shadow of a doubt as to the wisdom of the managers of the four districts in Northamptonshire who from the first have declined to employ any nurse unless she has had three years' training in a good general hospital. The complete financial success of their policy, and the increase of the nursing staff from one to four in seven years, may be set against all the arguments in favour of making shift with half-trained nurses.

### NURSES WANTED AT WOLVERHAMPTON.

The matron of the Queen Victoria Nursing Institution at Wolverhampton informs us that, in spite of her frequent advertisements for nurses, she cannot obtain the six fully-trained nurses she requires, though she offers a comfortable home, a good salary, and a bonus on reasonable conditions. We do not know that there is any special reason why nurses should avoid Wolverhampton. It is in the Black Country, or on the fringe of it, but it is a well-built town, there is charming country within an easy walk, Birmingham is only a short run, and the nursing can scarcely fail to be varied and interesting in character. As to the Institution itself, it was established in 1887, the Mayor is president, there is a thoroughly representative house committee, and the commodious building is in a good position. Some of the nurses who talk about going to South Africa or abroad might do better to try Wolverhampton.

### THEN AND NOW.

In the current number of the *Trained Nurse*, Dr. H. Warren White relates his personal experience as a patient during the spring of 1881 in Highgate Small-pox Hospital. They were of a very unpleasant character. The large ventilator over his bed was so wide open that while a snow storm was in progress, he felt the snow sift in on his face. Two of the patients close to him died and there were no screens to put round them. He continues, "There was no attention worth calling nursing as good nurses would not accept such a position. The nourishment handed round was thick slabs of bread and butter and a bowl of tea. My mouth and throat were very sore and I could not eat anything like this. After much begging I got some milk." If Dr. White had been in London during the recent epidemic of small-pox, and had paid a visit to one of the hospitals belonging to the Metropolitan Asylums Board, he would have found a very different state of things, namely, well-ordered wards, every comfort, plenty of nourishing food, and good nurses. In these days, partly because the conditions of nursing have been so greatly improved, and partly because among nurses the fear of small-pox has been reduced to a minimum, there is no difficulty in securing for sufferers from the disease all the care and attention they require.

### FORRES LEANCHOIL COTTAGE HOSPITAL.

There must be something radically wrong in the management of the Forres Leanchoil Hospital; but the position at the little institution in Morayshire, which was erected by the generosity of Lord Strathcona and others, is so shrouded in mystery that it is difficult to determine who is at fault. The dispute, which has been going on for nearly four years, appears to be concerning the rules of the hospital, and for 18 months Dr. John Berne has acted single handed at the hospital. In July last he intimated that he could not continue his attendance without injury to his private practice. On September 12th the managers announced that they had arranged with two other doctors to resume attendance at the hospital. Subsequently, three of the managers resigned, and the matron, Miss Agnes Reid, was verbally requested to do so. This is the more extraordinary because ever since her appointment in 1897 the managers have annually spoken of her in most glowing terms. On October 14th Miss Reid left the hospital, her place being temporarily filled by a staff nurse. We learn that Miss Reid demanded an apology for statements made against her by one of the doctors. An apology was sent, but she did not consider it sufficiently comprehensive. According to the matron's assertion, the managers offered her £20 to withhold this apology from the public. Here the matter rests for the present, but the most deplorable feature of the affair is that in September a visitor found the hospital without patients and the nurses unemployed.

### RESIGNATION OF THE MATRON OF SWANSEA HOSPITAL.

The matron of Swansea Hospital, Miss Margaret Bridger, has resigned in consequence of her acceptance of an important post in the Civil Hospital at Gibraltar. At a meeting of the Board of Management the chairman, Colonel Morgan, and Miss Dillwyn, all joined in paying a tribute to the good work done by Miss Bridger, and it was decided to advertise for a new matron at a salary of £75 per annum, rising by increments to £100.

### MARRIAGE OF MISS CHITTY.

The marriage of Miss Amy Robinson Chitty to Major-General Hassard of the Royal Army Medical Corps took place at St. Peter's, Eastbourne, on Thursday last week. Miss Chitty, who lately returned from South Africa, where as a member of the Army Nursing Service Reserve she did excellent service in nursing the sick and wounded soldiers, was trained at the Alfred Hospital, Melbourne, and commenced her nursing career in England as staff nurse at the General Hospital, Birmingham, where she was afterwards sister and operation nurse. She was subsequently sister at the Taunton and County Hospital, nurse-matron at the London Throat Hospital, and matron at the General Hospital, Stroud.

### NURSES' HOSTEL COMPANY.

The fifth annual report of the Nurses' Hostel Company, Limited, was adopted at the general meeting on Thursday last week. A dividend of 2½ per cent. was declared, and a small balance carried forward. The north block, it is stated in the report, accommodated 594 nurses in the course of the year, who had paid 1,791 visits, and in the six months since the south block was opened 323 nurses stayed in it, making an aggregate of 473 visits. The sum of £157 9s. 2d. has been withdrawn from the reserve to pay for improvements in the hot-water service in the original building. When the still numerous unlet rooms in the south block are occupied, there should be no necessity to diminish the reserve fund for purposes of this kind.

### NURSES FROM SOUTH AFRICA.

The following nurses have returned from South Africa during the week ending Wednesday: On the *Salamis*, Sisters H. Swain and E. C. R. Philp; on the *Dilwara*, Sisters M. L. Potter, K. Ward, and E. Fisher; on the *Sardinia*, Sisters E. H. Hay, Magill, E. Hamman, and M. C. Birt; and on the *Britannic*, Sisters E. T. Powdrell, N. P. Haine, and M. Robertson.

### CAMBRIDGE GUARDIANS AND DISTRICT NURSES.

An attempt to induce the Cambridge Board of Guardians to reduce the grant of £100 which was given last year to the District Nursing Association has, we are glad to notice, utterly failed. The member of the Board who wanted to knock off £20 of the amount, not only found no seconder, but was the means of eliciting very handsome and striking testimony from several of his colleagues to the exceedingly valuable character of the work done by the district nurses. One of the latter pointed out that for the donation of £100 the Board got the command of half a dozen nurses. He declared that if they did the work themselves, instead of tendering modest assistance, it would cost them from £300 to £400 a year.

### PROGRESS AT STOCKPORT.

The appeal of the Mayor of Stockport, twelve months ago, in the interests of the Sick Poor Nursing Association was so far successful that the report which was read at the annual meeting shows a balance in hand of £147. It is true that the number and variety of special contributions were exceptional, and included the proceeds of a concert, and a Fire Brigades' demonstration. But if the Mayor had not put forward a strong plea for the better support of the Association, some of these contributions might not have been made. There is no question of the excellence of the work done by the district nurses who, during the twelve months, paid 7,263 visits and attended 236 patients. But the Stockton Association labours, as we think, under the disadvantage of possessing two branches.

### PLAGUE AT A MISSION STATION.

The Zenana Bible and Medical Mission has received tidings of an outbreak of plague at their station in Malegaon, Western India. There have been two deaths; others are feared. Miss Harris, the missionary in charge, is doing her best to attend to the patients; but as, according to her account, the quarters of the mission are only a stone's throw from the Mohammedan quarter "where plague has been most fierce," we are afraid that she will badly feel the need of the help of trained nurses.

### CHELMSFORD GUARDIANS ON THE STOOL OF REPENTANCE.

According to Mr. Lunney, the Chelmsford Board of Guardians are now on the stool of repentance. In other words, they have under serious consideration a scheme for the improvement of the nursing in the workhouse infirmary. It includes the increase of the staff by the appointment of one trained nurse and two assistant nurses, a new time table for the nurses, and additions to their quarters. These proposals, which are certainly not too adequate, are, however, in advance of some of the members of the Board, who affirm that for "more than 25 years things have gone on very comfortably," and need not therefore be altered. This is the usual plea of reactionaries, and it is to be hoped that it will be over-ruled at Chelmsford.

### THE ROYAL UNITED HOSPITAL, BATH.

At the examination of nurses of the Royal United Hospital, Bath, the examiners expressed themselves "greatly pleased with the advance in the standard of the nurses, both in theory and practice, in spite of the status now required being nearly 30 per cent. higher than it was three years ago." Five third-year nurses presented themselves for their final examination, and the gold medal, presented annually by the Rev. E. Handley, was awarded to Nurse Reese. The silver medal was won by Nurse Mackay, and prizes for the second-year nurses, of whom there were eleven, by Nurse Drage and Nurse Johnson.

### CHELSEA INFIRMARY.

The first number of the *Chelsea Infirmary Nurses Journal*, which is admirably printed and arranged, contains an interesting letter to the matron from Miss Margaret Baldwin, giving an account of her experiences as nurse in a hospital at Foo Chow, South China, and a thoughtful article by Mrs. Hayward—formerly "Sister Elizabeth" at St. Barthomew's Hospital—on "Hospital Life in Perspective."

### STOCKTON NURSING HOME.

The new home at Stockton for the Stockton and Thornaby Nursing Association has been opened by Lady Londonderry. It was purchased a few months ago at a cost of £1,759, towards which the Association has provided £745 16s. 6d. There is still a balance of nearly £700 to be raised. As, however, the income of the Association has steadily risen till it is now £700 a year, this is not a very formidable sum to collect. An organisation which, as Lady Londonderry says, employs five highly-trained nurses constantly moving in and out of the homes of the poor of the town, attending in a year nearly 1,000 cases, has claims that cannot be resisted.

### NURSES AND THE ST. JOHN AMBULANCE ASSOCIATION.

At the annual distribution of medallions and certificates of the St. John Ambulance Association two recently-appointed probationers from the parish infirmary, Portsmouth, received their certificates. The chairman of the centre, Dr. Chas. Knott, in his speech, pointed out the good work of the society, and complimented the nursing staff on the practical assistance they had rendered.

### SHORT ITEMS.

The members of the Stock Exchange have subscribed upwards of £1,700 to Queen Victoria Jubilee Nurses' Institute, which brings the amount contributed by the City of London to over £3,200.—The Incorporated Society of Trained Masseuses, 12 Buckingham Street, Strand, W.C., will hold an examination on Tuesday and Wednesday, November 25th and 26th. Candidates wishing to enter for it must apply not later than November 8th.

### Lectures to Nurses on Anatomy.

By W. Johnson Smith, F.R.C.S., Principal Medical Officer, Seamen's Hospital, Greenwich.

### LECTURE XXIX.—THE SPECIAL SENSES.

*Vision.*—The eyeball is situated in a large cavity called the orbit in the upper part of the skeleton of the face, where it can move freely within a thick and soft investment of fatty tissue. In front it is protected against blows by the projecting upper margin of the orbit, and against the external air and bodies suspended in the air by two folds, *the eyelids,* made up each of skin, muscle, and a thin disc of cartilage; and by the attached eyelashes (fig. 63, *c*, *d*). Under cover of the outer portion of the upper margin of the orbit is the small lachrymal gland which serves usually to moisten the front of the eyeball by a scanty but continuous discharge of thin and clear fluid, and under casual circumstances, as external injury or painful mental emotions, pours forth this fluid in excess in the form of tears. Attached to the back of the eyeball is a thick and rounded nerve—the *optic nerve* (*s*)—which passes backwards to the base of the brain through a large hole, the *optic foramen,* at the apex formed by the converging walls of the

Fig. 63.—Vertical Section of the Eyeball and Eyelids.

*a*, crystalline lens; *b*, vitreous or posterior chamber, containing the vitreous humour; *c*, the upper, and *d*, the lower, eyelid; *e*, anterior chamber filled with aqueous humour; *f*, cornea; *g*, iris; *h*, posterior chamber; *i*, the upper, and *k*, the lower, *cul de sac* of the conjunctiva; *l*, the ciliary muscle; *m*, the suspensory ligament; *n*, section of the levator palpebræ superioris muscle; *o*, section of the superior rectus muscle; *p*, section of the inferior rectus muscle; *r*, optic disc; *s*, section of the optic nerve; *t*, sclerotic coat; *u*, choroid coat; *v*, retina.—From Eccles.

orbit. To the portion of the eyeball hidden within the orbit are attached six small muscles—four straight (*o*, *p*) and two oblique—which move the organ of sight in different directions.

The human eyeball is a hollow sphere about one inch in diameter in the adult, the anterior portion of which—about one-sixth of the whole circumference—bulges a little beyond the regular contour of the greater portion of the surface. This anterior and bulging portion of the wall of the eye, consists of a transparent membrane known as the *cornea* (*f*), reminding one of a round window, through which light is admitted into the interior of the globe.

The posterior and larger portion of the wall of the sphere is made up of three coats: (1) The sclerotic (*t*), forming the external and protecting case, is composed of tough and flexible fibrous tissue, the exposed part of which just around the cornea is commonly known as the "white of the eye"; (2) the *retina* (*v*) forming the inner coat is a membranous extension and expansion of the optic nerve, and the essential element in the construction of the organ of vision, as this structure alone is excited by the stimulus of light, and capable of converting for transmission through the optic nerve this stimulus into proper visual sensations; (3) an intermediate coat of a dark colour, the *choroid* (*u*) made up in part of a close network of very minute blood-vessels and in part of an inner layer of cells deeply stained by black pigment. This membrane with regard to its use or function corresponds to the black layer on the inner surface of a photographic camera.

The retina at the back, or *fundus* of the eye, where, by means of the ophthalmoscope it can be easily examined in the living subject, presents two spots of interest: the *yellow spot*, a circular elevation about one-twenty-fifth of an inch in diameter, with a yellowish margin, situated in the very centre of the retina, and at a short distance (one-eighth to one-tenth of an inch) to the inner side of this yellow spot the larger *white spot* or *optic disc* (*r*) which corresponds to the insertion of the optic nerve and to the entrance of blood vessels from without to the retina.

On examining in a drawing (fig. 63) or model a vertical section of the eyeball, we may observe that the curved cornea forms the front boundary of a small space—the *anterior chamber* (*e*)—which is separated from the much larger space behind—the *posterior chamber* (*b*)—by the transparent doubly-convex disc called the *crystalline lens* (*a*), and the movable curtain which gives the colour to the eye, known as the *iris* (*g*). The anterior chamber is filled by a clear watery fluid, the *aqueous humour* and the posterior chamber, by a compact and soft jelly termed the *vitreous humour.* The dark circle surrounded by the iris is the *pupil,* which under normal conditions enlarges or *dilates* in the dark, and becomes smaller or *contracts* when the eye is exposed to light. The iris loses its mobility, or the pupil becomes abnormally large or small under certain conditions of disease and injury of the brain and spinal cord, in certain cases of poisoning, and also under the influence of terror and other emotions. The lens is surrounded and kept in place by a thin membrane called the *suspensory ligament* (*m*), the outer portion of which is attached to a circle of longitudinal folds—the *ciliary processes*—formed by the anterior portion of the dark choroid coat of the eyeball. At the junction of the sclerotic coat and the margin of the cornea is a small circular muscle (*l*)—the *ciliary muscle*—which acts indirectly on the lens, and by altering its curvatures serves to adapt or *accommodate* the organ of vision to varying distances.

*Smell.*—To form some idea of the parts concerned in this special sense we must again examine the skull. If the cranial vault be removed and the inner surface of the base be exposed, we shall see in the anterior fossa between the roofs of the eye-sockets two narrow depressed surfaces of bone, separated the one from the other by a spike of bone, which from its resemblance to a cock's comb has been named by anatomists the *crista galli.* Each of these narrow strips of thin bone, which together form part of the roof of the nose, is perforated like a sieve with numerous minute holes which are arranged in longitudinal rows.

The olfactory nerves, the first of the twelve pairs of

### LECTURES TO NURSES ON ANATOMY.—*Continued.*

cranial nerves, rest on these perforated plates of bone, and from their lower surfaces give off a great number of very small nerve filaments which pass through the above-mentioned holes or foramina into the upper part of the nasal cavity on either side.

If we leave the base of the skull and look through the large nasal orifice in the midst of the bones of the face we shall see that the interior of the nose is divided into two lateral cavities by a septum or partition of thin bone, and that along the outer wall of each of these lateral cavities there are fixed three light convoluted bones placed one above the other. Of these bones, which are called turbinals, the upper one, which probably is out of sight, and the middle one, are both attached to a cranial bone called the ethmoid, of which the perforated plate of bone we have seen at the base of the skull also forms a part. To the mucous membrane covering these two bones and the upper part of the bony partition between the two nasal cavities, the minute branches of the olfactory nerves are distributed, and it is here in the upper part of the nose that the sense of smell is excited by the impact of odorous particles suspended in the inspired air. The *inferior turbinal*, which is freely exposed in the skull just beyond the entrance into the nasal cavities, is distinct from the other two turbinals with regard both to its associations and its function. It is not attached to the ethmoid, and as it receives no filaments of the olfactory nerve takes no part in the sense of smell.

*Taste.*—The chief organs of this special sense are derived from certain modifications of structure in the mucous membrane of the muscular and mobile organ we know as the tongue. The back of the palate and probably the pharynx and tonsils also, take some part in this sense which is a very complex one and in its operation is closely associated with the senses of smell, sight, and touch.

The mucous membrane covering the upper surface and the margins and tip of the tongue is not smooth like that of the lips and the interior of the mouth but is roughened by outgrowths varying in size, form and extent of distribution, which are termed *papillæ*. The most numerous of these which from their resemblance to very minute threads are known as *filiform papillæ* are crowded together over the upper surface of the tongue; whilst along the margins and at the tip of the organ are scattered somewhat larger projections which, from their swollen summits, are called *fungiform papillæ*. In addition to these there are eight or ten larger and more complicated outgrowths arranged in the form of a V quite at the back of the tongue. Each of these, which are called *circumvallate papillæ*, presents a broad central mass pitted on its free surface and surrounded by a circular depression, and beyond this by a circular elevation, the whole resembling a central fort with moat and outwork.

The portions of the tongue in which the perception of taste is most marked are the back part of the upper surface, and the sides and tip of the organ; just those parts that are occupied by the circumvallate and the clubbed or fungiform papillæ.

The sensory nerves of the tongue are derived from two sources: the fifth pair of cranial nerves, and the ninth pair, or glosso pharyngeal nerves. The questions of the relative parts taken by these cranial nerves in taste, and also whether the fifth nerve is, or is not to be regarded as the sole source of this special sense, are as yet unsettled.

### The Royal Red Cross.

BY A RECENT RECIPIENT.

From the age of 7 to 14 I thought that I had been very badly treated. Why was I only a girl? I had brothers older than myself, and they were all making their way at school, at college, and in the Army, and it seemed to me that there were endless ways in which they might distinguish themselves, but none in which I could do so. Then I read of the institution of the Royal Red Cross by Queen Victoria, and promptly decided that I would win it, and that the most probable way to do so was to train as a nurse. From 14 to 21 I studied every book I could get hold of on nursing and debated the advantages of the various training schools, deciding that I should prefer Guy's Hospital, but eventually entering for a preliminary year at the Hospital for Sick Children in Great Ormond Street because they would accept me at an earlier age than Guy's Hospital. From 21 to 28 I was hard at work "training." Then arose the question, Should I enter the Army Nursing Service as I had originally intended? But after very careful consideration I found that there were many things I should not care for in it, and that the position I then held as ward sister in a large London hospital gave me far more scope for useful work than I should find possible under the restricted sway which was allowed to an Army nursing sister. My visions of the Royal Red Cross had long before this disappeared, but had been replaced by the satisfaction of useful work in an absorbingly interesting profession.

### Volunteering for the War.

Then came the "Great Boer War," and every nurse longed to lend a hand. I did not volunteer at first. There were so many keen to go and I had many ties to England, and I thought that the war would soon be over; but as the months went by and one heard of the enteric playing such havoc amongst all ranks and of the hardships of the sick and wounded, it did not seem to be right to be living in comfort at home without at least offering one's services to the authorities. So one day I went to 18 Victoria Street and in the waiting-room a strange thing happened. I met an old friend who asked me what I was doing there. To which I replied, "Like everyone else, trying to get out to South Africa." Then he told me that he was offering to provide a hospital for the front. Would I go with it? Of course I would, and so in five minutes all the outlook for me was changed. The days flew by and I was appointed a member of the Army Nursing Service Reserve. I had to assist in the selection of the rest of the staff, to hand over my ward to another sister, to pay a flying visit to my home in the North, and in spare minutes to collect a suitable kit for abroad.

### On Active Service.

We sailed early in 1900, and then followed many months of hard work near the base; the cases were very bad, especially at first when, for military reasons, it was necessary to move the men down from the front, however bad they were. Often we had men brought in at the worst stages of enteric fever who had been shaking about in trains for a couple of days or even longer, and, of course, just then our orderlies, who were all St. John's Ambulance men, were of very little use for nursing purposes. They were excellent fellows, very hard-working and anxious to learn, and afterwards became very good nurses; but for the first few months it was very "hard-going" for the sisters to ensure that their patients were properly attended to. Later on I was given

### THE ROYAL RED CROSS—*Continued.*

work "at the front," and many times woke up in my tent to the tune of the Boer guns in the distance.

Mentioned In Despatches.

Now came the intelligence that I had received "a mention in despatches" by Lord Roberts, and later on that I was awarded the Royal Red Cross. I shall never be able to imagine why, as I had had no opportunity of ever doing anything remarkable. Of course we had worked hard; at times of pressure pretty well night and day; but so had a great many more, and really one could hardly help doing so when surrounded by so many desperately sick men and with so few trained people to attend to them. Months afterwards when I was home again in England the following letter came from the War Office:—"Madam, I am directed by the Secretary of State for War to acquaint you that the decoration of the Royal Red Cross sent out to Africa for presentation to you by Lord Kitchener has now been returned to this Department. I am to state that the King's pleasure will be taken as to the date of an investiture, and it is hoped that the opportunity will occur for presentation of the decoration in question to you by His Majesty." Next came the King's illness, which filled all our thoughts, and some months again passed before I received a further letter from the War Office as follows:—"The Under Secretary of State for War presents his compliments to Miss ——, and would inform her that the King will hold an Investiture of the Order of the Royal Red Cross on the 24th inst. If Miss —— has not yet received the decoration, and should she desire to be present at the Investiture, she should immediately communicate her wishes to the Under Secretary of State."

At Buckingham Palace.

Eventually, on October 24th, wearing my uniform and the South African war medal—which, by the way, had been sent to me by post—I found myself driving up to Buckingham Palace amongst the crowd of carriages filled with men in gorgeous uniforms, almost inclined to think that it would have been easier to keep calm if we had been driving up to the mouth of the Boer guns. It was a very large Investiture, and there were fourteen ladies to receive the Royal Red Cross. We were not kept waiting very long, but joined in the procession of officers who slowly made their way towards the ballroom, we sisters immediately following the one officer and two privates who received the Victoria Cross. I fear that from sheer nervousness some of us did not carry out the programme as we should have done. I know that we were supposed to kiss the King's hand, but it seemed to me that there was no time allowed to do it in, for His Majesty stooped as we curtseyed and pinned on the Royal Red Cross, and then with a gracious smile he half rose and took my hand and shook it. Then we backed away from the Royal presence, not quite sure if we were awake or dreaming. It was a pleasure to see among the ladies Georgina, the Dowager Countess of Dudley, who did so much for the disabled officers and sisters when they returned from the front, helping them back to health and strength; the new matron-in-chief of the Army Nursing Service, from whom we hope for great things in the re-organisation of the service; the matron of the Royal Free Hospital, who gave such valuable aid on the Committee of the Army Nursing Service Reserve; and others whose work one had heard of at the front. The war is over, and we trust that it has taught us many lessons. At any rate it "gave us all a chance," and amongst my many treasured relics, including Queen Victoria's chocolate box, none will be so much valued as my Royal Red Cross.

### Supporting a Patient in a Bath.

### EXAMINATION QUESTIONS FOR NURSES.

The question for October was as follows:—"State what arrangements you would make for supporting a patient with the least possible fatigue during treatment which consists of immersion in a bath for many hours at a time."

First Prize.

If the whole of patient's body is to be immersed I should improvise a sort of hammock with a strong sheet or sail canvas if obtainable, in which to sling the patient in the bath, using an ordinary firm pillow with a mackintosh cover under pillow-case, or an air-cushion as a support for the patient's head. Securing the ends of hammock with some strong cord and tying on to some nails driven either into the floor close to head and foot of bath or into walls, or else secure them with heavy weights on the floor, having that part of hammock under patient's head well raised out of the water. In the event of the bath being fixed against the wall as is often the case in private houses, I should then place the patient in a strong sheet or blanket put lengthwise across the bath, placing weights on the sheet on that side of bath next to wall, and the same on the floor other side, with support for the head as in hammock. Hip baths are useful when it is considered desirable to immerse that part of the body only, in which case I should place a folded rug or blanket with a long narrow-shaped pillow or roll of something soft to fit comfortably into nape of patient's neck, a pillow under the feet, and a support for the arms in the shape of stools or books with pillows on the top to bring them to height of bath on either side.—Hopeful.

Second Prize.

I should first get a large blanket, or strong sheet blanket preferred; if a large one cannot be obtained, two small joined together could be used, but they must be firmly joined, then spread it in the bath, laying the ends well on the floor; allow the blanket at the foot to touch the bath, then raise it up at the head so as to form a hammock, then on the ends of blanket at the head stand two buckets of water, or scuttle of coal on either side, so as to counteract the weight of patient. A large folded bath towel could be placed under patient's head to raise it a little; at the foot of the bath put a small wooden box or hot-water bottle, or two bricks wrapped in a small towel or new piece of house flannel, and put close against foot of bath, then bring patient's feet to rest against it so as to prevent patient from slipping down. Place a blanket over the bath up to patient's neck.—"Niggs."

The Merits and Defects of this Month's Papers.

The answers this month are on the whole good, a decided advance on those to the same question which engaged our attention some three or four years ago, though there is still room for very considerable improvement. The chief defect is the failure to remember all points connected with the subject—they state what they consider to be the salient points and fondly imagine that an ordinary intelligence will grasp the rest. This is not unnatural, but you must all remember that if you aspire to the higher walks of the nursing profession you will be called upon to teach others, and a clear, telling way of imparting your knowledge is of the first importance. It is not only necessary for a ward sister to be herself a first-class nurse, but she must be able to make others so.

On the whole "Hopeful" of Torquay's answer is the best. I state her locality because three "Hopefuls" sent in papers. "Niggs," the winner of the second prize, writes a very good paper, short, practical, and to the point. I see capa-

### SUPPORTING A PATIENT IN A BATH—*Continued.*

bilities in a nurse who utilises humble buckets of coal or water, which are to be had in every cottage. She would have gained the first prize, but alas! for one little defect—she says, "allow the blanket at the foot to touch the bath." This is a mistake—if the feet could touch the floor of the bath they would quickly become sore, soddened already by long immersion, and as it would be the only part of the body in contact with a hard substance and the tendency of all weights is to bear downwards, the pressure would shortly become unbearable.

### Honourable Mention.

This is gained by "R. R." "Hope" and "Nurse M." It would be an improvement if nurses would use more distinctive pseudonyms. "Nurse M." may be anybody, and "Hopefuls" "Inexperienced" and "Pros." are in their thousands.

"Nurse M.'s" plan of attaching her hammock by means of a cord under the beaded rim is good. "Hope" devotes too much space to a hip bath and not enough to total immersion.

"R. R.'s" paper is the least good, but in some respects it gives evidence of considerable care and thought, as when she speaks of pouring in fresh hot water at the side to avoid causing nervousness to the patient.

### Question for November.

Imagine yourself called to assist a doctor in a lonely cottage miles from a town. The case is one of simple fracture of the leg. Not having been told it is a case of fracture the doctor has brought no splints. What would you do to help him in this difficulty?

### EXAMINATION QUESTIONS FOR OUR NURSES IN THE COLONIES AND ABROAD GENERALLY.

The question was the same as that set last month to nurses in the United Kingdom.

### First Prize.

A firm, comfortable support and a great sense of security to the person to be immersed is furnished by a sling of stout jean, made hammock fashion, with three loops at one end, a broad webbing strap and buckle at the other, and also at the sides. The three loops, in the case of a fixed bath, are slung on to the taps, and the "hammock" is made taut by the straps from the sides being brought over the edges of the bath and buckled underneath, while the one from the end is threaded through it and secured.

The shoulders and head of the patient should be supported by firm pillows covered with mackintosh, over which cotton pillow-cases may be placed, as indiarubber becomes very sticky and unpleasant to the skin if hot water be used; while an upward and forward position is maintained by a pile of sandbags, similarly encased, used as a foot-rest.

The bath may then be covered with fracture boards and blankets, serving the dual purpose of retaining the heat of the water and avoiding the risk of draught to the patient. This sling can be modified and used with baths of almost every size and shape. "Nina."

### Only One Thoroughly Good Answer.

As time goes on we hope that our nurses across the seas will respond in larger numbers and to better purpose.

"Nina's" answer is excellent. She is the only nurse either at home or abroad who has thought of utilising the taps which are found on most, though not on all baths. She does not make it quite clear why there should be sandbags at the feet, as the invalid would be sufficiently supported by the hammock, but presumably it would be to give the patient something to push against. He would not require this as there would be no slipping down in a properly swung hammock and the sandbags are best omitted.

### Defects in other Papers.

It is unadvisable to put shirt or nightdress on a patient who is undergoing this treatment. Several nurses say they should have it if they wish it, but that is a mistake, consider the chilly process of taking it off, and in the case of a helpless patient, the impossibility of doing so without considerable delay. Patients, they state, dread exposure, but a good nurse will avoid that, and her previous manner and arrangements will prevent such fears.

### Question for April.

How would you feed a patient suffering from enteric fever, whilst the nourishment ordered by the doctor is to be strictly liquid? N.B.—The question supposes that the medical man in attendance gives only general orders and leaves the nurse to exercise her discretion.

### Rules.

The competition is open to all. Answers must not exceed 500 words, and be written on one side of the paper only. The pseudonym, as well as the proper name and address, must be written on the *same* paper, and *not on a separate sheet.* Papers may be sent in for fifteen days only from the day of the publication of the question. All illustrations strictly prohibited. Failure to comply with these rules will disqualify the candidate for competition. Prizes will be awarded for the two best answers. Papers to be sent to "The Editor," with "Examination" written on the left hand corner of the envelope.

N.B.—The decision of the examiners is final, and no correspondence on the subject can be entertained.

In addition to two prizes honourable mention cards will be awarded to those who have sent in exceptionally good papers.

### Note the only Alteration Necessary for Nurses in Foreign Lands.

Instead of the sentence referring to the competition being open for fifteen days, all papers for the April competition must therefore reach this office by March 20th, 1903.

The Examiner.

### Presentations.

Preston Royal Infirmary.—Miss C. Stuart Cameron, who is leaving Preston Royal Infirmary to take up the duties of home sister at Sheffield Union Infirmary, has been presented with a gold anointing spoon from the matron, a silver-mounted photograph frame from the assistant matron, and a timepiece from the sisters and nurses.

Newcastle Nurses' Home.—The staff of the Nurses' Home, Granville Road, Newcastle, have presented the matron with a gold lever watch, and the assistant with a gold brooch set with sapphires and diamonds in commemoration of the Coronation.

Royal Southern Hospital, Liverpool.—A very interesting ceremony took place on Friday at the Royal Southern Hospital, Liverpool. In presence of a full medical board and several members of the general committee, the president of the hospital, Mr. Wm. Adamson, presented to Miss Gordon, on her retirement from the hospital management, a purse of one hundred guineas, subscribed by the general committee and medical board. Miss Gordon has been 19 years matron of the institution, in the management of which she has given entire satisfaction. She is one of a family of nurses, her elder sister having been 12 years matron at St. Thomas's Hospital, London, and her younger sister 10 years matron at Charing Cross.

### Wants and Workers.

Nurse Williams, Broadway, Worcestershire, would pay postage and would be grateful for copies of The Hospital passed on to her as soon as done with.

### The County Nursing Associations Affiliated to Queen Victoria's Jubilee Institute.

BY AMY HUGHES, SUPERINTENDENT.

THERE is no doubt in the minds of those interested in the work of nursing the sick poor in their own homes as to the desirability of every nurse thus employed being fully trained, *i.e.*, that she should have full hospital, district, and maternity training.

At present, however, this seems impossible. All those concerned in the training of nurses know the difficulty of obtaining candidates to meet existing requirements. Even if money were forthcoming to enable every nursing centre in England to maintain fully-trained nurses, the supply of suitable women would be quite inadequate to the demand.

Moreover, unfortunately, money is frequently not forthcoming. Also, nurses accustomed to the responsibility of numerous serious cases are often naturally disinclined to settle in rural districts, where work is, as a rule, "dull," the conditions of life different to those in which they have for the most part been trained, and where the opportunities for the exercise of their special powers are few and far between. Such nurses naturally tend to gravitate towards towns and populous districts.

These facts have had to be faced and dealt with by those responsible for the nursing of the poor in the scattered country districts of England, and the "village nurse," employed by county associations affiliated to the Queen's Institute, is the outcome of long and careful consideration of the peculiar needs and difficulties of the case, and what has so far been done may be considered as at any rate a step towards the solution of this most complicated problem. In the first place, the village nurse is a fully-trained midwife, and as such is specially fitted for rural work, where a large proportion of cases are either directly, or indirectly, connected with child-birth.

In addition to this, the village nurse is instructed in such general details of nursing as fit her to deal with chronic and ordinary cases of illness. This knowledge is gained in the homes of the poor, under fully-trained nurses, so that she learns from the beginning to accommodate herself to the surroundings of her patients.

She is not trained for the special nursing of complicated cases, and can only be employed to attend them at the discretion of the medical man in charge of the case. The village nurse is not placed in a district, and there left without guidance and without direction. The Queen's Institute provides that all village nurses employed by county associations affiliated to it, shall be under the supervision of a fully-trained Queen's nurse, who, as county superintendent, pays more or less frequent visits to all nurses employed in the county, and who gives special help and advice to the village nurses, thus carrying on their education. The county superintendent can also be referred to in all cases of emergency. The position thus held by village nurses is an important one.

Trained as midwives, they render services which cannot be over-estimated to the women of our country places. They in no way interfere with the practice of the medical men in this work. Each local committee is urged never to allow its nurse to undertake midwifery unless it is the wish of the doctor that she should do so; but by their training these nurses learn how to act in emergencies, and are also better able to nurse such cases intelligently under the directions of the medical men.

Under the Midwives Act, they will hold an increasingly important position, being prepared to replace the self-taught, well-meaning, but often ignorant, women who now attend so many poor mothers in their confinements. Drawn for the most part from the districts in which they work, and accustomed to the country from childhood, they face bad roads, dark nights, and winter storms with a confidence unknown to the imported nurse. They live contentedly on the funds which can be raised locally for them, and find friends amongst their neighbours.

They are capable of following out simple instructions in cases of ordinary illness, and do well in more difficult ones under the kindly help of the doctors and the supervision of the county superintendent.

It is hoped that some will go on for full training at the completion of their agreement with the County Associations, which will be gladly afforded them by the Queen's Institute.

There is no wish to consider or class these village nurses as trained nurses; but as midwives, with some knowledge of the general principles of nursing, their services are an inestimable boon to the mothers and infants of our land, and a comfort and solace in times of sickness to those who, without them, must be left to the kindly but unskilled attentions of relations and friends.

### Appointments.

[No charge is made for announcements under this head, and we are always glad to receive, and publish, appointments. But it is essential that in all cases the school of training should be given.]

ASTON WORKHOUSE INFIRMARY, GRAVELLY HILL, BIRMINGHAM.—Miss Mary Ann Camamile has been appointed head night nurse, and Miss Julia Lund charge nurse. Miss Camamile was trained at the Central Sick Asylum, Cleveland Street, London, and has since been head nurse at Lambeth Infirmary, and at Cardiff Union Infirmary. Miss Lund was trained at Keighley Union Infirmary, and has since been nurse at the Convalescent Home, Cotham, Redcar.

CANNOCK WORKHOUSE INFIRMARY.—Miss Catherine M. Henfield has been appointed head nurse. She was trained at Aston Workhouse Infirmary.

CHELSEA HOSPITAL FOR WOMEN.—Miss Mary A. Butt has been appointed sister, and Miss Bessie Wingfield and Miss Ada Stewart assistant nurses. Miss Butt was trained at Leicester Infirmary, where she has since been staff nurse in the women's operation wards with theatre attached, and temporary night sister. Miss Wingfield was trained at St. Mary's Hospital, Paddington, and the Chelsea Hospital for Women. Miss Stewart was trained at the Middlesex Hospital and the Chelsea Hospital for Women.

CHERITON ISOLATION HOSPITAL.—Miss A. Effie Dolbell has been appointed matron. She was trained at the Kent and Canterbury Hospital, and the North-Eastern Hospital, London. She has since been staff nurse at Fulham Infirmary, London, and Dorset County Hospital; charge nurse at Park Fever Hospital, and head nurse at the Isolation Hospital, Colchester.

COTTAGE HOSPITAL, BRIXHAM.—Miss Kathleen Disney has been appointed matron. She was trained at the London Hospital, where she was afterwards staff nurse and night sister. Subsequently, she was matron at Boston Hospital, temporary matron at Taunton and Somerset Hospital, and matron at the Royal Infirmary, Preston.

FYLDE HOSPITAL, MOSS SIDE, NEAR LYTHAM.—Miss Sara Daly and Miss Olive Roche have been appointed charge nurses; both were trained at Monsall Fever Hospital, Manchester.

Hospital for Consumption, Brompton.—Miss Mabel Newill has been appointed home sister. She was trained at King's College Hospital, London, and has since been night sister at the Seamen's Hospital, Greenwich, and ward sister at University Cottage Hospital.

Iver, Langley, and Denham Cottage Hospital.—Miss Dorothy Salt has been appointed nurse-matron. She was trained at the Children's Hospital, Derby, and the Hospital and Dispensary, Rotherham. She has since been charge nurse at the Fever Hospital, Scarborough, and on the staff of the York Home for Nurses, York, for four years. Subsequently she joined the staff of Princess Christian's Trained Nurses, Windsor, where she has acted as private nurse, and also for 14 months as Queen's district nurse.

Johnson Hospital, Spalding.—Miss Janet Cooke has been appointed matron. She was trained at the Guest Hospital, Dudley; and at the General Hospital, Nottingham; and has been sister at the Royal Infirmary, Sheffield; theatre sister at the same institution; night superintendent at the Stanley Hospital, Liverpool; assistant matron at the Ilkley Hospital and Convalescent Home; and matron of the Montagu Cottage Hospital, Mexborough.

Lewisham Infirmary.—Miss E. Gertrude Hobbs has been appointed sister. She was trained at Guy's Hospital, and has since been staff nurse at the Royal South Hants and Southampton Hospital, charge nurse at the Northern Fever Hospital, and sister at the Women's Hospital, Harrow Road, London. Miss Hobbs holds the L.O.S. certificate.

Liverpool Sanatorium, Frodsham.—Miss Kate Parry has been appointed sister. She was trained at Mill Road Infirmary, Liverpool, and has since been staff nurse at the Brompton Hospital for Consumption and Diseases of the Chest.

London Lock Hospital and Rescue Home, Harrow Road, W.—Miss E. W. Clements has been appointed night sister. She was trained at the Hospital for Women, Soho, and the Royal Infirmary, Sheffield.

Royal Southern Hospital, Liverpool.—Miss Sproule has been appointed matron. She has been for more than twenty years nurse, and latterly sister, at the Royal Southern Hospital.

St. Peter's Hospital, Henrietta Street, London, W.C.—Miss Florence Furley has been appointed Matron. She was trained at the West London Hospital, and has since been night superintendent and day sister at St. Mark's Hospital, City Road, London. She has also taken the matron's holiday duties during the last six years.

Sheffield Union Infirmary.—Miss Christina Stuart Cameron has been appointed home sister. She was trained at Dumfries and Galloway Royal Infirmary and has since been charge nurse at Cardiff Hospital, sister at Hartlepool Hospital, and sister at Preston Royal Infirmary.

### To Nurses.

We invite contributions from any of our readers, and shall be glad to pay for "Notes on News from the Nursing World," or for articles describing nursing experiences, or dealing with any nursing question from an original point of view. The minimum payment for contributions is 5s., but we welcome interesting contributions of a column, or a page, in length. It may be added that notices of appointments, entertainments, presentations, and deaths are not paid for, but that we are always glad to receive them. All rejected manuscripts are returned in due course, and all payments for manuscripts used are made as early as possible after the beginning of each quarter.

### Everybody's Opinion.

### QUEEN ALEXANDRA'S IMPERIAL MILITARY NURSING SERVICE.

"One Who Waits" writes: Having read with great interest the various articles which have appeared in your paper concerning the new rules of the Q.A.I.M.N.S., may I be allowed to express my opinion on the subject of a staff nurse's pension? If the number of staff nurses to enter the service is to be 200, receiving as their pay £35 a year, after ten years' service, if disabled, their pension would be £10 10s. a year—not enough to keep them in clothes. It is all very well to say that a nurse will be promoted to the rank of sister after two years, but how can 200 nurses get promotion when there are to be 27 matrons and 50 sisters? There would be over one hundred nurses who could not possibly get promotion and would have to go on to the end, with what inducement? Of course, nurses must remember that they will lose all experience in women and children's work and even of people over the age of 40, and, therefore spoil their chance of procuring any good appointment in a civil hospital. Now a word as to uniform. While any non-commissioned officer is allowed to wear mufti when off duty if he wishes, is it not a little unfair that a sister will not be allowed to decide for herself? There is no such strict rule in any large civil hospital and why should there be in a military hospital?

"A Reserve Sister" writes: In reading over the rules and regulations of the "Queen Alexandra's Imperial Military Nursing Service" and learning the duties delegated to matrons, sisters, and staff nurses, one wonders at the somewhat startling rules laid down for their obedience. Are matrons to take the places of quartermasters and staff sergeants? A matron or sister should have the cleanliness of the patients and wards under her direct supervision; but the responsibility of the entire equipment is a very different thing. Only those who have worked in a military hospital know what it means and how one orderly makes up his full equipment when loss or damage occurs by taking or borrowing from another so that he escapes paying for the loss. To what extent of loss in equipment is the matron or sister to be held responsible, and out of whose pocket is it to come? What civil matron is held responsible for all the equipment of the wards under her charge and has to pay for loss through theft which she cannot account for? Equipment does, and will always continue to, go astray even in the best regulated wards. Under the present regulation each orderly is held responsible for his ward equipment and any extra linen he may have on charge in that ward; therefore, if a sheet, towel, or even corkscrew is lost he simply goes to another ward and helps himself. Is this what our sisters and nurses are to come to—one taking from the other if she happens to be short of an article rather than be considered incompetent to take care of the things? Then, sisters are to be held responsible for the kits of the patients, which will involve them in more trouble than meets the eye. When kits come to be claimed by patients who have been too ill to give or take a receipt for the articles given in for them when entering the hospital there are very often endless questions as to what they had and what they had not, and surely it is not a woman's work to quibble about such things, and more likely than not to have to attend a court of inquiry about the matter. It is not probable that any sensible woman knowing what she is about will sign a declaration stating that she is willing to undertake the responsibility for such things as these without first finding out to what extent she may be held liable in paying for lost and stolen equipment and making up deficient kits. One can understand the sister being held responsible for the linen in the wards; that is supposing she has not more than two wards, but in a large military hospital where each sister has three or four wards, and is held responsible for the linen in them all, and also for knives, forks, spoons, mugs, etc., I wonder when she will find time to go into the details of making out statements concerning breakages and loss which this responsibility involves in military work. Again, when fresh patients are admitted, generally speaking they arrive in batches of from 20 upwards to 100, and when portioned off to their different wards, orderlies and ward-masters are told off to draw their equipment in the shape of sheets, pillow cases, hospital clothes, knives, forks, spoons, etc., each man having the regulation allowance given to him individually. His kit has to be handed into stores, and if he is able to do so himself he does it, and receives a receipt for the things, but if he is unable to walk this is done by the orderly for him. In future the duty is to become the work of the sisters and nurses. When, in that case, are their other duties to be performed? The chief fault in military hospitals so far has been the inadequate number of sisters to do the proper nursing of the patients, and it seems only reasonable to expect when that number is increased, by adding staff nurses, that the nursing should have better supervision; but how can this be accomplished when the sisters are doing quartermasters' and staff sergeants' work, and thereby decreasing the number of men employed? As you have announced, there are to be 27 matrons, 50 sisters, and 200 nurses on the new nursing staff, and such first-class orderlies and non-commissioned officers as are selected. When one takes into consideration the number of hospitals throughout the country to be staffed from these, and compares them with civil hospital staffs which, I believe, the ruling authorities are trying to emulate, and then add the duties of staff sergeants, there does not seem much room left for the reforms aimed at. In comparing the salaries of nurses and orderlies it will be seen that a staff nurse, who is to be a well-educated lady of good family and a three-years' trained nurse, will have to instruct the third-class orderlies, who are recruits. For this she is to be paid about a sovereign more than the boys (they are little more) whom she is teaching. The first-class orderly, who, as far as his work is concerned, ranks with a nurse who has just completed her three years' training, is to receive a salary as high as the staff nurse will receive after two years' further service. Then we come to the corporal, who receives £44 3s. 9d. and the sergeant £57 17s. 6d., the sister, who is to do the work previously theirs, as well as her own, simply receiving £37 10s. rising to £50. One asks, Is it fair to any woman that such a state of things should be permitted? It may be urged that the man is paid more so that he may keep a wife and family, but has not the woman her old age also to provide for? On this matter I do not agree with the sister who wrote "Army Nursing, Past and Future," and who seems to think the salaries are all that could be desired or expected.

### THE FUTURE OF THE ASYLUM NURSE.

"One Interested in Mental Work" writes: If all doctors were like Dr. David Hunter, medical superintendent of the West Ham Lunatic Asylum, it might be hoped that, at no very distant date, mental nurses and mental nursing might occupy a much higher position in popular opinion than they do at the present time. Unfortunately, Dr. Hunter may be taken as rather the exception who proves the rule than as a type of the medical profession in this particular matter. Some years ago it was probably true that those to whom the personal care of the insane was confided were not of an order characterised by ultra refinement and delicacy of feeling, and that a certain coarseness and lack of sensibility came to be associated in the popular mind with mental nursing. That state of things has passed, or is passing, and that the change is not making greater progress is perhaps due in some measure to the want of moral support and co-operation from the medical profession towards women of education and refinement who have made a speciality of this branch of nursing. For certainly upwards of five years the care of the mentally afflicted has been made a special study by women who have brought to bear upon their work the advantages of a cultivated intelligence and the sympathy of a wider educational scope and inherited refinement. The ranks of mental nurses are reinforced to-day from a very different class to that from which they were formerly recruited, and it is to be hoped that a still increasing number of refined and educated women will devote themselves to such an important branch of the nursing profession. Its peculiar difficulties call for the tact and discriminating sympathy rarely found save in natures of refinement and sensibility. It is chiefly due to the want of interest shown by the doctors, and to the fact that not only do they not demand nurses of education and sound general and special training for their mental patients, but if they get such nurses they appear not to know how to treat them, that a larger number of educated women do not qualify for this work, which to many must present special psychological interest. Doctors can do much to modify the erroneous popular opinion of mental nurses and their work. In many houses it is from the doctor that the patient and the patient's friends take their tone towards the nurse. This is specially true of mental cases. If the doctor would give to the nurse the support of his authority and courteous co-operation, the family are likely to follow his lead, the nurse is enabled to do her best for the patient, and friction, often the result of "trifles light as air," is probably avoided. Unhappily, there is not merely a negative line of conduct on the part of the doctors in this matter, but occasionally positive discourtesy towards nurses of mental patients which would hardly be shown to those in charge of medical or surgical cases. Here is a case in point. A nurse with special mental training was nursing a lady whose maid was also in attendance. It was necessary for the patient to be examined. When the doctor came he requested the nurse to fetch the maid to assist him with the examination. This was quite unnecessary as the patient made no objection to the nurse who was thus ignored by the doctor. The effect of such conduct on the part of a doctor to a nurse is most detrimental both as regards her influence with the patient and her relations with the patient's friends. It is such acts that retard the "elevation of the standard" of mental nursing; and the possibility of being subjected to what is certainly an indignity is most discouraging to those who might be inclined to adopt this particular line of nursing.

### VILLAGE NURSES IN CUMBERLAND.

"A. B.," whose first letter appeared in our issue of October 4th, writes: In reply to the Countess of Lonsdale, I have no wish to make any comparison between the village and the hospital-trained nurse, as I cannot say from experience which possesses the greatest "smattering" of district nursing, but the majority of the people in this district prefer the latter, as the nurse is able to decide from her diagnosis of a case whether medical assistance is needed or not. She is also able to take all normal maternity cases alone. All this is a great saving of expense to the poor. The village nurse of a few months' training cannot possibly compete with her more experienced sister in these respects, and under the rules of the Cumberland Nursing Association, I believe, is not expected to do so. The Countess of Lonsdale says it is the great wish of the Cumberland Nursing Association that a fully-trained nurse should be employed wherever it is possible to support one. The population of this scattered district according to the last census is 911. At a public meeting it was decided to obtain the services of a more experienced nurse than the village nurse as it was thought that for a little more money such a nurse would be likely to be of greater use and benefit to the working people of the district. This has proved to be the case. The committee have been fortunate in always securing a most skilful nurse, and as the majority of the people in the district are subscribers they are able to cope with the expense. Yet the Cumberland Nursing Association keep a village nurse in this same district of 911 people, and gives a grant of money to assist the local funds, whilst some of the members or subscribers of the local branch have done their level best to drive the experienced nurse out of the district.

### INDIAN ARMY NURSING SERVICE.

"C. Evelyn Stroughill" writes: The name of the new member of this service mentioned in your last issue is Stroughill, not Stronghill.

### TRAVEL NOTES AND QUERIES.

France for Good Accent (M. S.).—No, I do not think the terms you mention at all cheap. It is over 7s. per day. The equivalent roughly is 8fr. 50c., and one expects very good accommodation for that in France for a prolonged stay in a boarding house. If the rooms were very good, and the cooking excellent and plentiful, it was no doubt worth 8fr. 50c.; but the amount is beyond what the generality of our correspondents could pay. Many thanks all the same for information, it comes in usefully sooner or later.

### Echoes from the Outside World.

### The King and His People.

THE Royal Progress through London which took place on Saturday was a most successful function. The weather was brighter and warmer than it had been on the date of the Coronation in August, and the route being nearly eight miles in length, a very large concourse of people were able to witness the pageant. Everywhere the King and Queen were greeted with much enthusiasm, and both appeared remarkably well, His Majesty smiling repeatedly at the cheers of the multitude; whilst the Queen, wearing a toque of cream and gold resembling a crown, looked very sweet and dainty. The State coach left Buckingham Palace at noon. In Trafalgar Square an address was received from the London County Council, and briefly acknowledged by the King; close to Norfolk Street another address from 17 mayors of the Northern Boroughs was offered by the Mayor of Westminster; and then at Temple Bar the Lord Mayor surrendered the sword of the City to the Royal visitor.

At the Guildhall their Majesties were received by the Lord Mayor and the City Corporation in an ornate pavilion erected in the Guildhall yard. Six hundred guests sat down to lunch. The ménu was in the form of a very pretty booklet bound in vellum; the tablecloth with an Imperial design had been specially woven for the occasion; and the floral decorations consisted of great baskets of ferns and trailing plants and flowers hung from the roof, whilst the table was ornamented with Alexandra orchids and Royalty carnations. All the viands were cold except the turtle soup, and the wines were of the choicest vintages. The first toast was "His Majesty the King," and Madame Albani sang the solo of the National Anthem. At half-past two their Majesties resumed their Royal Progress, crossed over London Bridge to South London, where they received an address of welcome at St. George's Circus, Borough, and went by way of Westminster Bridge and Whitehall to Buckingham Palace, which was reached about 3.40. Amongst others who witnessed the procession were the Boer generals, Botha, De Wet, and De la Rey, who, by invitation of the Chairman of the County Council, occupied a stand in Trafalgar Square; the *Terrible* men who were in the Square and in Pall Mall; and the Balaclava veterans, who were to be seen at a window in Fleet Street.

A Thanksgiving Service for the recovery of the King was held in St. Paul's Cathedral on Sunday, which was attended by the King and Queen and many other members of the Royal Family. The sermon was preached by the Bishop of London, who made a touching allusion to the fact that it was the second time that the King had appeared in the City Cathedral to return thanks for recovery from most serious illness. The rain came down at frequent intervals, but many spectators lined the route both on the Embankment and along Holborn.

On Monday morning the King reviewed the Guards upon their return from active service in South Africa. All the regiments were included, with the exception of the 2nd Scots Guards, who, with what was called their "customary bad luck," did not land at Southampton in time to be present. The men were in bearskins and scarlet, and in the rear were nine companies of Reservists and time-expired Guardsmen in plain clothes. The King was punctual to the moment, and arrived on horseback as the clock struck eleven. He wore the uniform of the Grenadier Guards, Lord Roberts that of the Irish Guards, whilst the Prince of Wales and the Duke of Connaught were attired respectively as a General and a Colonel of the Scots Guards. The Queen and Princess Victoria were in a carriage at the saluting base, and in another carriage were the Duchess of Connaught and her daughters. Amongst other onlookers of note was Lord Methuen, supporting himself on a stick. After having inspected the force, the Guards marched past the King and Queen. They were followed by the Reservists, who, though in plain clothes, were greeted with very hearty cheers. Then the King rode forward and made a stirring speech. He welcomed the warriors home, he said, as their Sovereign and their Colonel-in-Chief. He had followed their conduct with much interest since they left these shores, and could only mete out to them that praise which was their due. He confessed that he would even be glad to think that in his younger days he had served in their ranks, though he regretted that, unlike his brother, he had never seen active service in the field. His Majesty concluded, "I can only say it is a proud day for me to have inspected the Brigade of Guards." Then the men raised their bearskins on high, and cheered a mighty cheer for their King.

### Queen Victoria's Friend.

LADY BIDDULPH, a devoted friend of Queen Victoria, died on Thursday last week at her residence, Henry the Third's Tower, Windsor Castle. She had entered her seventy-ninth year, and up to a short time before her death she had been staying at Bournemouth, but was brought back to Windsor a week ago. On the day that she passed away the King went down from London in his covered motor-car to Windsor especially to visit her, showing how highly he holds in esteem all those who were attached to his mother. Lady Biddulph has always been closely associated with the Royal Family, and Queen Victoria alluded to her and her family in "More Leaves from the Journal of a Life in the Highlands." Up to six years ago Lady Biddulph held the office of First Lady in Waiting to Princess Henry of Battenberg, who has frequently visited her during her illness.

### Mr. Chamberlain to Visit South Africa.

IT was announced on Monday that, with the King's approval, the Secretary of State for the Colonies will leave England in the latter part of this month for South Africa in order to examine on the spot the problems presented by the termination of the war and the settlement of affairs in the new colonies. Mr. Chamberlain hopes to have the opportunity of conferring with the representatives of all the different interests concerned, and to consider their views as to future policy. The announcement of his impending visit, which will include the Cape, Natal, the Orange River Colony, and the Transvaal, and will probably extend over a period of about three months, has been received with enthusiasm both at home and in South Africa. It is regarded as an event of the utmost importance, and it is believed will have the most excellent results. Mr. Chamberlain will be accompanied on his tour by his wife, and at the King's desire will make the voyage in one of His Majesty's ships.

### The Somaliland Rising.

FORTUNATELY, the anxiety felt last week about Colonel Swayne and his force in Somaliland has been dispelled. The gallant officer arrived at Bohotle without being attacked during retirement, and reports that the wounded are doing well, including the two officers, the wound of one being slight, and the other, though injured severely in the shoulder, being able to ride. But the orders warning the Punjab regiments to be in readiness to leave India are not to be cancelled, because the Mullah is probably spending his time in augmenting his forces, and is consequently in a more important position than at any stage of his career. He and his followers are now in undisputed possession of a large tract of difficult country, and it will need a large, and practically a new, expedition to take successful measures to put down the rising.

### For Reading to the Sick.

ALL SAINTS' DAY.

O Lord, to Whom the spirits live
Of all the faithful passed away,
Unto their path that brightness give
Which shineth to the perfect Day.
Light Eternal, Jesu blest,
Shine on them, and grant them rest.

Bless Thou the dead which die in Thee,
And make their painful labours cease,
O purge them from impurity,
And give them everlasting peace.
Light Eternal, Jesu blest,
Shine on them, and grant them rest.

We must learn to realise the City, and its Citizens, the Society, the Company, in which our true life is thrown: giving some time, quietly and calmly, to take in these unseen realities, so as to gain a truer apprehension of the world invisible. To us, in this present world, it is invisible; and yet, in one sense, it is as present as this visible world. It is a real world, now going on, into which any of us may, any day, be called to enter. It is around us now. The majority of men are there already; and most of us will also be there, before 50 years are over.—*Bishop Webb.*

Already "our citizenship is in heaven." Already we are "fellow-citizens with the saints and of the household of God." Already we belong to the "Jerusalem which is above, which is the mother of us all."

And the spiritual greatness of our calling sheds its heavenly glory and its beauty over the oft darksome and toilsome lot, in this world, of many a lowly Christian life that is lived in the light of that faith, under the observation of scarcely any eyes but those of God and His Holy angels. "Such honour have all His Saints;" and, perhaps, most of all those who are His "secret ones."—*P. G. Medd.*

Is it not a joy to think that when we clasp our friends again in heaven, and look back with them upon the past, it will be to see it, not as we have felt it, but as it is; to take, not man's view, but God's; to know, and know together, that the dark scenes were dark with light too bright for mortal eye, the sorrow turning into dearest joys when seen to be the filling up of Christ's, who withholds not from us His own crown, bidding us drink of His cup, and be baptised with His baptism, and saying to our reluctant hearts, "What I do thou knowest not, but thou shalt know hereafter"?—*J. Hinton.*

They worship in the courts of paradise
As in a dim and vast cathedral aisle
Lit up with golden sunshine like the smile
Of God; and evermore around them rise
Sweet clouds of incense with the prayers of saints;
The Eucharists of sanctuaries on earth
(Dear precious home that saw their blest new birth),—
The whispered words of the loved soul that faints
For sorrow, as in loneliness it strives
To picture their exceeding joy and peace.
O hallowed atmosphere of purest prayer,
Bathed in whose light our troubled mumurings cease!
Communion of the saints, how blest to share
The angel's offering, incense of our lives!

*Helen Douglas.*

### Notes and Queries.

**The Editor is always willing to answer in this column, without any fee, all reasonable questions, as soon as possible.**

**But the following rules must be carefully observed:—**

**1. Every communication must be accompanied by the name and address of the writer.**

**2. The question must always bear upon nursing, directly or indirectly.**

**If an answer is required by letter a fee of half-a-crown must be enclosed with the note containing the inquiry, and we cannot undertake to forward letters addressed to correspondents making inquiries. It is therefore requested that our readers will not enclose either a stamp or a stamped envelope.**

*Abroad.*

(29) Will you kindly tell me to whom I should apply for a post on the staff of a nursing association in Paris? What is the address of the English Hospital there?—*Sister.*

There are no nursing associations in Paris. The address of the English Hospital is The Hertford British Hospital, rue de Villiers-Lavallois-Perret, Paris.

Will you kindly tell me if there are any homes for trained nurses in Durban, Natal, or Capetown, South Africa?—*I. M. M.*

The Victoria Nurses' Institute, Capetown, Cape Colony. But the Lady Superintendent already has more applications than she can reply to.

*Hospital Training.*

(30) Is it possible for me to get a three-months' training in a fever hospital, as I have had a year's general training in a cottage hospital? Then, with my general and fever training, what post would you advise me to apply for in South Africa?—*Faith.*

You are not eligible for any post as a trained nurse until you possess a three-years' certificate from a general hospital at which there is a resident medical officer. We should advise you to train properly before you emigrate, as there are plenty of fully-qualified nurses on the spot.

*District Nurse.*

(31) I am a clergyman's daughter and am anxious to obtain a few months', or a year's training, so as to be able to nurse the poor in the parish. I am not able to pay a premium nor to go where no salary is given.—*M. E. H.*

You cannot get any training and receive a salary without taking a three-years' course of training. You might, however, write to the Mildmay Mission Hospital and Dispensary, Austin Street, Bethnal Green, E.

I am desirous of becoming a district nurse, but have not had hospital training. I am a little over forty years of age, of medium height, and enjoy good health.—*E. H.*

You will have some difficulty in being trained for a district nurse at your age. Probably your best plan would be to write to the Secretary of the Affiliated Benefit Nursing Associations, 12 Buckingham Palace Road, S.W.

*Seaside.*

(32) Will you kindly tell me if Southend-on-Sea is a healthy place, and if warm baths can be obtained there for a patient suffering from rheumatism?—*M. M. H.*

In a matter of such importance as choosing a locality for invalids, it is necessary to consult a good medical man.

*Septic Infection.*

(33) 1. Would it be possible that a mother could show signs of septic infection three days after confinement in consequence of the nurse having shared her bed during menstruation? 2. What book on domestic medicine would you recommend for home use for one who understands medical terms a little?—*L. G.*

1. A maternity patient should sleep alone. It is quite possible that the infection came from the cause mentioned. 2. Dr. Braidwood's "The Mother's Help and Guide to the Domestic Management of her Children." Price 2s. The Scientific Press.

**Standard Nursing Manuals.**

"The Nursing Profession: How and Where to Train." 2s. net; post free 2s. 4d.

"A Handbook for Nurses." (Illustrated). 5s.

"Nursing: Its Theory and Practice." (New Edition.) 3s. 6d.

"Nursing in Diseases of Throat, Nose, and Ear." 2s. 6d.

"Surgical Ward Work and Nursing." (Revised Edition.) 3s. 6d. net; post free, 3s. 10d.

"Art of Massage." (Second Edition.) 6s.

"Elementary Physiology for Nurses." 2s.

"Elementary Anatomy and Surgery for Nurses." 2s. 6d.

"Practical Handbook of Midwifery." 6s.

"Mental Nursing." 1s.

"Art of Feeding the Invalid." 1s. 6d.

All these are published by the Scientific Press, Ltd., and may be obtained through any bookseller or direct from the publisher 28 and 29 Southampton Street, London, W.C.

## Figures and Tables

**Fig. 63. f1:**